# Bimetallic Nickel Cobalt Sulfide as Efficient Electrocatalyst for Zn–Air Battery and Water Splitting

**DOI:** 10.1007/s40820-018-0232-2

**Published:** 2019-01-09

**Authors:** Jingyan Zhang, Xiaowan Bai, Tongtong Wang, Wen Xiao, Pinxian Xi, Jinlan Wang, Daqiang Gao, John Wang

**Affiliations:** 1 Key Laboratory for Magnetism and Magnetic Materials of MOE, Key Laboratory of Special Function Materials and Structure Design of MOE, Lanzhou University, Lanzhou, 730000 People’s Republic of China; 2 School of Physics, Southeast University, Nanjing, 211189 People’s Republic of China; 3 Department of Material Science and Engineering, National University of Singapore, Engineering Drive 3, Singapore, 117575 Singapore; 4 Key Laboratory of Nonferrous Metal Chemistry and Resources Utilization of Gansu Province and The Research Center of Biomedical Nanotechnology, Lanzhou University, Lanzhou, 730000 People’s Republic of China

**Keywords:** (Ni,Co)S_2_ nanosheet arrays, DFT calculations, Zn–air batteries, Water splitting

## Abstract

The development of efficient earth-abundant electrocatalysts for oxygen reduction, oxygen evolution, and hydrogen evolution reactions (ORR, OER, and HER) is important for future energy conversion and energy storage devices, for which both rechargeable Zn–air batteries and water splitting have raised great expectations. Herein, we report a single-phase bimetallic nickel cobalt sulfide ((Ni,Co)S_2_) as an efficient electrocatalyst for both OER and ORR. Owing to the synergistic combination of Ni and Co, the (Ni,Co)S_2_ exhibits superior electrocatalytic performance for ORR, OER, and HER in an alkaline electrolyte, and the first principle calculation results indicate that the reaction of an adsorbed O atom with a H_2_O molecule to form a *OOH is the potential limiting step in the OER. Importantly, it could be utilized as an advanced air electrode material in Zn–air batteries, which shows an enhanced charge–discharge performance (charging voltage of 1.71 V and discharge voltage of 1.26 V at 2 mA cm^−2^), large specific capacity (842 mAh g_Zn_^−1^ at 5 mA cm^−2^), and excellent cycling stability (480 h). Interestingly, the (Ni,Co)S_2_-based Zn–air battery can efficiently power an electrochemical water-splitting unit with (Ni,Co)S_2_ serving as both the electrodes. This reveals that the prepared (Ni,Co)S_2_ has promising applications in future energy conversion and energy storage devices.

**Electronic supplementary material:**

The online version of this article (10.1007/s40820-018-0232-2) contains supplementary material, which is available to authorized users.

## Highlights

- Bimetallic nickel cobalt sulfide (Ni,Co)S_2_ nanosheet arrays were demonstrated as a multifunctional catalyst for OER, HER, and ORR.
- First principle calculations were performed to probe the rate-limiting step, which involves the formation of *OOH from HO^−^ on the (Ni,Co)S_2_ surface.
- A water-splitting system was designed with the (Ni,Co)S_2_ serving as both cathode and anode, and a Zn–air battery cathode electrocatalyst.

**Electronic supplementary material:**

The online version of this article (10.1007/s40820-018-0232-2) contains supplementary material, which is available to authorized users.

## Graphical abstract

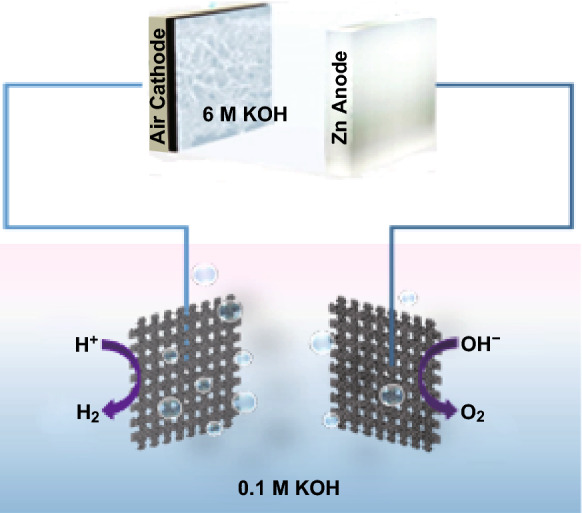

## Introduction

The ever-worsening environmental issues and non-renewability of fossil fuels have stimulated extensive investigations for the development of sustainable energy in future energy conversion and storage technology [1–3]. The high-rate oxygen reduction or evolution reaction (ORR or OER) and hydrogen evolution reaction (HER) at lower overpotentials are of great importance to the enhancement of energy utilization rate and output power in these green energy systems. At present, the bottleneck of both water-splitting technologies and rechargeable metal–air batteries is the availability of highly efficient and durable electrocatalysts. Zn–air batteries have the merits of high theoretical energy density, environmental friendliness, and high safety for the next-generation energy storage systems [4, 5], where its development is still hampered by a low working voltage owing to the sluggish rate of ORR/OER [6, 7]. Here, HER, which is a crucial electrochemical reaction in water splitting and requires highly efficient electrocatalysts, is equally important [8, 9]. Pt-based materials exhibit excellent catalytic efficiency for HER and ORR, while Ru- and Ir-based materials are the best electrocatalysts for OER reactions [10–12]. However, their high scarcity, high cost, and insufficient long-term stability are limiting the large-scale commercial applications [13, 14]. Therefore, earth-abundant, durable, and highly efficient trifunctional (ORR, OER, and HER) electrocatalysts are urgently required [15, 16].

As a class of low-cost alternatives, transition metal-based materials, such as transition metal phosphides [17, 18], oxides [19–21], sulfides [22, 23], selenides [24, 25], nitrides [26, 27], borides [28, 29], hydroxides [30, 31], and others [32–34], have attracted overwhelming research interests recently. In particular, transition metal sulfides, such as CoS_2_ and NiS_2_, are considered a group of low-cost and eco-friendly electrocatalysts for ORR, OER, and HER owing to their high electrocatalytic activity, high stability, and cost-effectiveness [35–37]. Substitution of the transition metals with other dopants (such as V, Mn, and Cu) has been proved to enhance their electrocatalytic performance because of the synergistic effects among the metallic atoms [38–40]. Caban-Acevedo et al. [41] recently demonstrated that the replacement of S atom by P atom in CoS_2_, forming CoPS, could alter the electronic structure and dramatically enhance the HER performance. Liang et al. [42] also revealed that their bimetallic NiCoP nanostructures show superior catalytic activity toward both HER and OER in alkaline media compared to monometallic Ni_2_P. Although similar efforts are expected to be made for the bimetallic NiCoS, compared with the monometallic counterparts, challenges exist in the design of multifunctional catalysts.

As is known, both CoS_2_ and NiS_2_ have the same crystal structure, and the chemical nature and atomic radius of Ni and Co atoms are very similar, which would enable the formation of bimetallic NiCoS. In this work, we present a detailed study on the synthesis of single-phase bimetallic nickel cobalt sulfide (denoted as (Ni,Co)S_2_) nanosheets by the hydrothermal process and subsequent post-sulfuration. The resulting (Ni,Co)S_2_ shows the desired trifunctional electrocatalytic activities in OER, ORR, and HER as an electrocatalyst, and therefore has promising potential as a cathode in Zn–air batteries and water-splitting catalysis. In addition, it demonstrates excellent OER activity with an overpotential of 270 mV at 10 mA cm^−2^ and a notable outstanding potential difference (Δ*E* = *E*_*j*=10_–*E*_1/2_) between *E*_1/2_ for ORR and *E*_*j*=10_ for OER of only 0.79 V, thus outperforming many of the bifunctional electrocatalysts. The air electrode made of (Ni,Co)S_2_ nanosheets exhibited superior performance in both primary and rechargeable Zn–air batteries, showing a specific capacity of 842 mAh g_Zn_^−1^ at 5 mA cm^−2^, a high and stable open circuit potential of 1.48 V, a large peak power density of 152.70 mW cm^−2^, and excellent cycling stability without any decrease in polarization even after 480 h. The rechargeable Zn–air batteries using (Ni,Co)S_2_ as the cathode could efficiently power an electrochemical water-splitting unit catalyzed by the (Ni,Co)S_2_ nanosheets grown on a carbon cloth for both OER and HER, thus demonstrating its potential as an integrated green energy system.

## Experimental

### Synthesis of NiS_2_, CoS_2_, and (Ni,Co)S_2_

#### Preparation of Precursors

Precursors for (Ni,Co)S_2_ were synthesized on a carbon cloth by modifying a reported procedure [39]. First, 1.5 mmol NiCl_2_·6H_2_O, 3.0 mmol NH_4_F, 7.5 mmol (NH_2_)_2_CO, and 1.5 mmol Co(NO_3_)_2_·6H_2_O were dissolved in 50 mL de-ionized water. Then, 16 mL of the solution was transferred to a 23 mL PTFE-lined stainless steel autoclave containing the substrate leaning against the autoclave wall. The sealed autoclave was heated at 110 °C for 5 h. After cooling, the substrate was taken out, washed with water and ethanol, and dried in an oven at 60 °C for 30 min. The precursor of NiS_2_ or CoS_2_ was prepared by the same above-mentioned process, except without the addition of Co(NO_3_)_2_·6H_2_O or NiCl_2_·6H_2_O, respectively.

#### Thermal Conversion

A carbon cloth covered with the as-grown precursor was placed in the center of a fused silica tube in a tube furnace equipped with gas flow controllers. An alumina boat containing 10 mmol of sulfur powder was placed at the furthest upstream position within the reactor tube. The tube was then purged three times with argon gas and maintained at 101.3 kPa under a steady flow of Ar carrier gas (99.999%) at 25 sccm (standard cubic centimeter per minute). The temperature of the furnace was ramped to 500 °C and held for 60 min. After cooling under Ar flow, the sample was removed and rinsed with CS_2_ (99.9%) for 10 min, then washed with ethanol, and dried in an oven at 60 °C for 1 h.

### Preparation of Electrocatalyst Ink

The catalyst ink was typically made by dispersing 10 mg of the catalyst and 10 mg of carbon black (Vulcan XC72) in 50 mL petroleum ether, and then dropped them on a carbon cloth. After drying, 18 mg of catalyst, 90 μL Nafion-117 solution, and 4410 μL *N*, *N*-dimethylformamide (DMF) were added into a 10 mL container and ultrasonicated for 30 min.

### Calculation Details

The DFT calculations were performed by Vienna ab initio simulation package (VASP). The standard generalized-gradient approximation (GGA) in the form of the Perdew–Burke–Ernzerhof (PBE) exchange model was used. The energy cutoff for the plane-wave basis set and the convergence threshold to obtain the wave functions were 400 and 10^−5^ eV, respectively. Further, 3*d* electrons of Ni were treated using the GGA + *U* method with a *U*_eff_ (*U*–*J*) of 5.76 eV. Ionic relaxations were conducted until all the force components became < 0.02 eV Å^−1^. For the density of states (DOS), the Brillouin zone is represented by the set of 5 × 5×5 k points for geometry optimizations. A rectangular supercell of size 11.00 × 11.00 Å^2^ was used to calculate the OER activity with active sites on the (100) surface.

## Results and Discussion

**Fig. 1:**
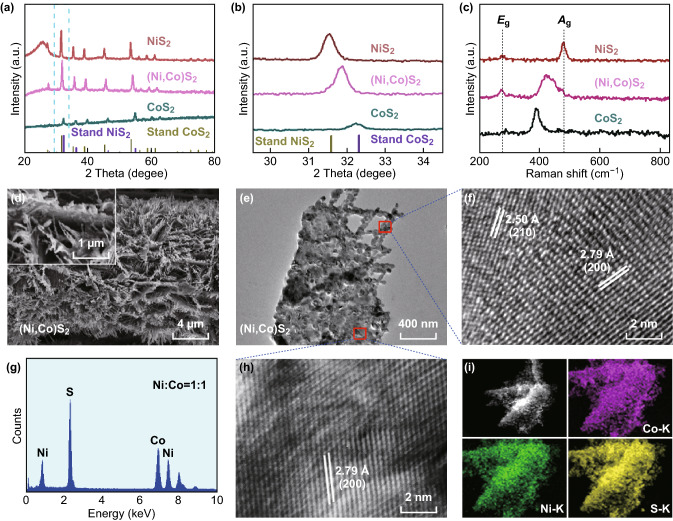
**a** XRD spectra of (Ni,Co)S_2_, NiS_2_, and CoS_2_. **b** High-resolution XRD spectra with 2 theta angles ranging from 30.5° to 33.5°. **c** Raman spectra of (Ni,Co)S_2_, NiS_2_, and CoS_2_. **d** SEM image of (Ni,Co)S_2_. The insert is high-magnification SEM image. **e** TEM image, **f**, **h** HRTEM images, and **g** EDS spectrum of (Ni,Co)S_2_. **i** EDS mapping images of Ni, Co, and S elements in (Ni,Co)S_2_

The phase structure of each sample was measured by X-ray diffraction (XRD). As shown in Fig. 1a, the XRD patterns of both NiS_2_ and CoS_2_, which were fabricated as reference samples, correspond to a cubic structure with a *Pa*-*3* space group (JCPDS No. 11-0099 for NiS_2_ and No. 41-1471 for CoS_2_) with lattice constants of *a* = *b* = *c* = 5.567 Å and *a* = *b* = *c* = 5.538 Å, respectively. The XRD pattern of (Ni,Co)S_2_ is consistent with those of NiS_2_ and CoS_2_ with its diffraction peaks located between those of NiS_2_ and CoS_2_, which can be clearly seen from the magnified area of the XRD pattern with the 2*θ* angles ranging from 30.5° to 33.5° (Fig. 1b). This confirms the formation of a single-phase crystal structure, where Co and Ni are alloyed in the bimetallic compound structure. Raman spectroscopy was employed to further confirm the formation of single-phase (Ni,Co)S_2_. For the monometallic samples, distinct peaks are observed at about 479 cm^−1^ for NiS_2_ and 391 cm^−1^ for CoS_2_ corresponding to the out-of-plane *A*_g_ vibrational mode (Fig. 1c) [43, 44]. For (Ni,Co)S_2_, the peak of *A*_g_ vibrational mode is located at 425 cm^−1^ between those of NiS_2_ and CoS_2_, indicating that the atomic vibration in (Ni,Co)S_2_ is a unified whole; this concurs with the XRD result. In addition, an obvious peak at 276 cm^−1^ is observed for all the three samples, which corresponds to the in-plane vibration mode of *E*_g_ for the cubic structure [45, 46]. The morphology was studied by both scanning electron microscopy (SEM) and transmission electron microscopy (TEM). Figure 1d shows that the as-synthesized (Ni,Co)S_2_ has the morphology of cactus-like nanosheets growing uniformly on the carbon cloth, which are nanoplates with some nanowires at the edges. It is not a combination of two morphologies but is a single morphology, which can be confirmed by subsequent high-resolution TEM (HRTEM) analysis. In contrast, NiS_2_ has nanoplate morphology, while CoS_2_ has nanowire morphology with a diameter of 50–100 nm (Fig. S1). The TEM images shown in Fig. 1e further confirm the formation of the observed nanosheet morphology with nanowires grown at the edges. Figure 1f, h shows the HRTEM images of the nanosheet and nanowire regions of (Ni,Co)S_2_, where lattices with spacings of 2.79 and 2.50 Å can be assigned to the (200) and (210) planes of (Ni,Co)S_2_, respectively. These lattice spacing values are between those of NiS_2_ and CoS_2_ (Fig. S2), which further reveals that (Ni,Co)S_2_ is a single-phase structure. The energy-dispersive X-ray spectrum (EDS) shows that (Ni,Co)S_2_ has a Ni/Co atomic ratio of 1:1 (Fig. 1g). The element mapping images in Fig. 1i show that Ni, Co, and S elements are uniformly distributed in the selected area.

**Fig. 2:**
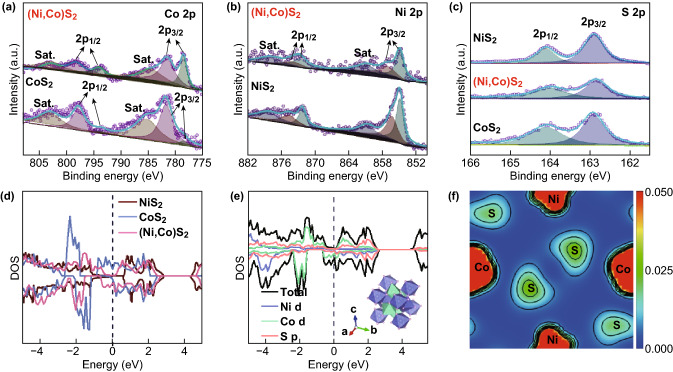
High-resolution XPS spectra of **a** Co 2*p*, **b** Ni 2*p*, and **c** S 2*p*. **d** Density of states (DOS) curves of (Ni,Co)S_2_, NiS_2_, and CoS_2_. **e** Partial density of states (PDOS) curves of (Ni,Co)S_2_. Insert shows the optimized crystal structure of (Ni,Co)S_2_. **f** Charge density distribution of (Ni,Co)S_2_

To further examine their composition and valence state, X-ray photoelectron spectroscopy (XPS) measurements were performed. The wide spectrum of (Ni,Co)S_2_ reveals the presence of Ni, Co, and S elements (Fig. S3). The Co 2*p* spectrum in Fig. 2a shows the main peaks of Co 2*p*_3/2_ and Co 2*p*_1/2_ along with their satellite peaks. For CoS_2_ and (Ni,Co)S_2_, two peaks appear at 778.5 and 781.6 eV, which belong to Co 2*p*_3/2_ and indicate the presence of Co^2+^ [47]. The Ni 2*p* spectrum (Fig. 2b) shows 2*p*_3/2_ and 2*p*_1/2_ doublets due to spin–orbit coupling. The Ni 2*p* spectra of both NiS_2_ and (Ni,Co)S_2_ show two peaks at 854.7 and 856.3 eV corresponding to Ni 2*p*_3/2_, and a satellite peak at higher binding energies [48]. The S 2*p*_1/2_ and S 2*p*_3/2_ peaks for these three samples are located at 164.1 and 162.8 eV, respectively, corresponding to (S_2_)^2−^ (Fig. 2c) [49]. The binding energies of Ni 2*p* and Co 2*p* in (Ni,Co)S_2_ show no obvious shift compared with NiS_2_ and CoS_2_, revealing that the Ni and Co atoms are uniformly distributed in the crystal structure. Thus, we demonstrated the formation of single-phase (Ni,Co)S_2_ in our case. Figure 2d shows the calculated DOS of (Ni,Co)S_2_, NiS_2_, and CoS_2_. It can be seen that (Ni,Co)S_2_ shows metallic nature with more electron-occupied states at the Fermi level, while CoS_2_ shows metallicity and NiS_2_ shows semiconductor characteristic (bandgap = 0.6 eV). As shown by the schematic in Fig. S4 and the inset of Fig. 2e, (Ni,Co)S_2_ has a cubic crystal structure, with Ni atoms replacing half of the Co atoms, adopting the CoS_2_ structure. The partial density of states (PDOS) curves of (Ni,Co)S_2_ shown in Fig. 2e indicate a strong hybridization of Ni 3*d*, Co 3*d*, and S 2*p*, which combined with the charge density distribution results (Fig. 2f), suggest an outstanding electrical conductivity of (Ni,Co)S_2_. The PDOS of NiS_2_ and CoS_2_ are shown in the supporting information for comparison (Figs. S5, S6).

**Fig. 3:**
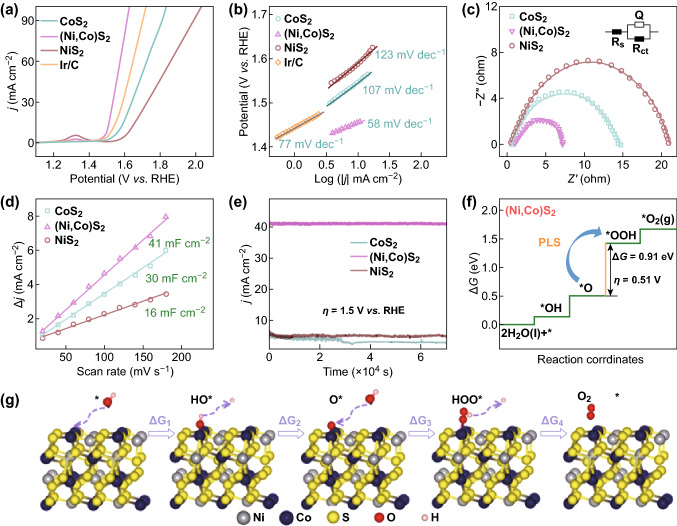
**a** Polarization curves of (Ni,Co)S_2_, NiS_2_, CoS_2_, and Ir/C (20% Ir) at 5 mV s^−1^ in 0.1 M KOH. **b** Tafel slopes obtained from the corresponding polarization curves. **c** EIS of (Ni,Co)S_2_, NiS_2_, and CoS_2_. The inset is an analogue circuit diagram. **d** *C*_dl_ obtained by cyclic voltammetry at different scan rates. **e** *I*–*t* curves at 1.5 V versus RHE for 7 × 10^4^ s. **f** Schematic of Gibbs free energy changes in the four elementary steps during OER on (100) surface of (Ni,Co)S_2_. **g** Proposed 4-step OER path presented using the model of (100) surface of (Ni,Co)S_2_

The performance of (Ni,Co)S_2_ in electrocatalytic oxygen evolution is evaluated using a three-electrode configuration in 0.1 M KOH solution, where NiS_2_, CoS_2_, and commercial Ir/C catalysts are used as the control samples. The polarization curve is firstly obtained by linear voltammetry scanning (LSV). The original LSV curves for OER are shown in the supporting information (Fig. S7). After converting by the method detailed in the supporting information, the standard polarization curves are obtained. As shown in Fig. 3a, the onset overpotential of (Ni,Co)S_2_ is 1.47 V, which is lower than that of NiS_2_ (1.57 V), CoS_2_ (1.50 V), and commercial Ir/C (1.48 V). For NiS_2_ and (Ni,Co)S_2_, there is a visible oxidation peak at about 1.32 V, which is similar to that previously reported for Ni-based catalysts [48]. Upon deducting the thermodynamic water decomposition voltage of 1.23 V [50], (Ni,Co)S_2_ shows an initial overpotential of 240 mV. When the current density reaches 10 mA cm^−2^, the overpotential of (Ni,Co)S_2_ is 270 mV and compares favorably to 410, 350, and 310 mV for NiS_2_, CoS_2_, and Ir/C, respectively. As shown in Fig. 3b, the Tafel slope of (Ni,Co)S_2_ is 58 mV dec^−1^, which is smaller than that of NiS_2_ (123 mV dec^−1^) and CoS_2_ (107 mV dec^−1^) and is close to that of Ir/C (77 mV dec^−1^). The reduced Tafel slope indicates that (Ni,Co)S_2_ exhibits a faster dynamics in the OER process [51]. Electrochemical impedance spectroscopy (EIS) was further employed to understand the interfacial electron transport between the electrolyte and catalyst at 1.45 V versus RHE from 10 kHz to 0.1 Hz. The curve fitting and equivalent circuit analysis results of the EIS data agree well with the (RQR) model. In Fig. 3c, *R*_s_ is the solution resistance (~ 3 Ω) and *R*_ct_ is the charge transfer resistance. A lower *R*_ct_ corresponds to a faster electronic transmission [52]. As illustrated in Fig. 3c, the EIS of (Ni,Co)S_2_ shows the smallest radius corresponding to the minimum *R*_ct_ value and indicates a faster reaction rate than those of NiS_2_ and CoS_2_. The double-layer capacitance (*C*_dl_) is obtained by cyclic voltammetry (CV) performed at different scan rates (in the range of 20–180 mV s^−1^, Fig. S8) to evaluate the electrochemical active sites [53]. Figure 3d shows that the *C*_dl_ of (Ni,Co)S_2_ is 41 mF cm^−2^, which is larger than the *C*_dl_ of CoS_2_ (30 mF cm^−2^) and two times the *C*_dl_ of NiS_2_ (16 mF cm^−2^); this reveals that there are more electrochemical active sites in (Ni,Co)S_2_ for OER. The synergistic effect of Ni and Co in (Ni,Co)S_2_ activates new active sites, increasing the electrochemical active surface area (EASA). To further assess the OER catalytic rates, the turnover frequencies (TOFs) of the three electrocatalysts were estimated, assuming that all the metal ions in the electrocatalysts were catalytically active (Fig. S9) [54]. As shown in Fig. S9, at an overpotential of 1.55 V versus RHE, the TOF of (Ni,Co)S_2_ is 3.02 s^−1^, whereas the TOFs of CoS_2_ and NiS_2_ are 0.65 and 0.22 s^−1^, respectively. This suggests that (Ni,Co)S_2_ has the fastest rate for OER catalysis. The OER parameters of the three catalysts and Ir/C are listed in Table S1 for comparison. As shown in Fig. 3e, even after testing for 70,000 s at 1.5 V, the current density of (Ni,Co)S_2_ remains at 40 mA cm^−2^, revealing the outstanding stability of (Ni,Co)S_2_ in OER compared with the degenerated current densities of NiS_2_ and CoS_2_. A comparison of the OER performance of (Ni,Co)S_2_ with that of other typical catalysts is shown in Table S2. For comparison, the OER polarization curves of the physically mixed CoS_2_ and NiS_2_ (named CoS_2_ + NiS_2_) were also examined, which reveals that its performance is in between those of CoS_2_ and NiS_2_ (Fig. S10). Besides, the structure and morphology after the long cycling test and the structural evolution process are shown in Figs. S11–13. The diffraction peaks of the cycled (Ni,Co)S_2_ are similar to those of the fresh (Ni,Co)S_2_, indicating that it retains its phase even after prolonged tests. The surface of the nanosheets is constantly corroded during repeated charge and discharge tests, resulting in a coarse surface and some oxidation state. The entire OER progress can be summarized in four elementary reaction models consisting of three key intermediates: *OH, *O, and *OOH (Fig. 3g) [55]. Each elementary step releases H^+^ cation and electron. It is crucial for the intermediate to have an appropriate Gibbs free energy. Figure 3f shows the Gibbs free energy (Δ*G*) diagram for the (100) surface of (Ni,Co)S_2_ with correlative intermediates at different reaction steps. It can be seen that the third step is the potential limiting step (PLS), where an adsorbed O atom reacts with a H_2_O molecule to form a *OOH. The overpotential (*η*) calculated by DFT calculations is 0.51 V for *O + H_2_O (l) ⇌ *OOH + H^+^ + e^−^, which is smaller than that of pure CoS_2_ (*η* = 0.54 V) and NiS_2_ (*η* = 2.00 V) at the (001) surface (Figs. S14, S15). The calculated results are consistent with the above experiment results, indicating that bimetallic (Ni,Co)S_2_ can be a better electrocatalyst than monometallic CoS_2_ and NiS_2_.

**Fig. 4:**
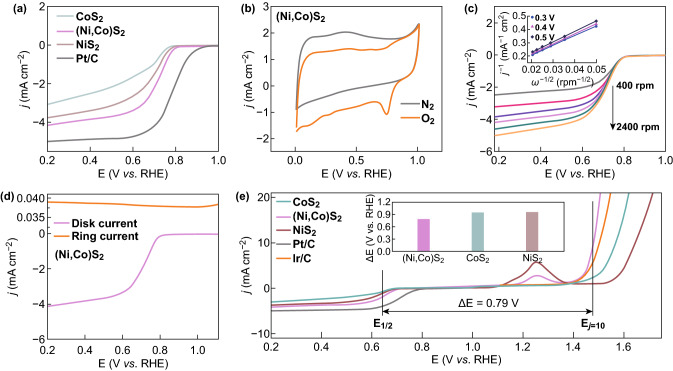
**a** ORR polarization curves of (Ni,Co)S_2_, NiS_2_, CoS_2_, and Pt/C (20% Pt) at 2 mV s^−1^ in 0.1 M KOH at 1600 rpm. **b** CVs of (Ni,Co)S_2_ in O_2_ and N_2_-saturated 0.1 M KOH solution. **c** ORR polarization curves of (Ni,Co)S_2_ at different rotation rates from 400 to 2400 rpm. The inset figure is the *K*–*L* plots. **d** RRDE polarization curves of (Ni,Co)S_2_ at 1600 rpm. The ring electrode was polarized at 1.5 V at a scan rate of 2 mV s^−1^. **e** Bifunctional electrocatalytic activities of various catalysts toward both ORR and OER

Oxygen reduction activities were studied to determine the suitability of (Ni,Co)S_2_ as a bifunctional electrocatalyst for both ORR and OER. It was examined with a rotating disk electrode (RDE) in 0.1 M aq. KOH electrolyte at room temperature. As shown in Fig. 4a, (Ni,Co)S_2_ shows the highest onset overpotential of 0.82 V, while it is 0.79 and 0.76 V for CoS_2_ and NiS_2_, respectively. The limiting current densities were measured as 4.2, 3.0, and 3.7 mA cm^−2^ for (Ni,Co)S_2_, CoS_2_, and NiS_2_, respectively, at overpotential of 0.20 V. The half-wave potential of (Ni,Co)S_2_ (0.71 V) is slightly smaller than that of Pt/C (0.78 V) but higher than that of CoS_2_ (0.63 V) and NiS_2_ (0.68 V). The physically mixed sample CoS_2_ + NiS_2_ was also measured. The results show that its ORR performance is between that of CoS_2_ and NiS_2_, which illustrates the importance of (Ni,Co)S_2_ as a single-phase bimetallic catalyst (Fig. S16).

The CV scan results are shown in Fig. 4b. The curve measured in a N_2_-saturated electrolyte solution is smooth, indicating no oxygen reduction reaction. However, in the O_2_-saturated electrolyte solution, a sharp cathodic peak appeared at 0.75 V, revealing the occurrence of an ORR. Under the same test conditions, the oxygen reduction peaks of NiS_2_ and CoS_2_ are 0.74 and 0.67 V, respectively (Fig. S17). To explore the reaction mechanism of oxygen reduction, LSV curves with various speeds (from 400 to 2400 rpm) were measured, and the results shown in Fig. 4c indicate that the current density increases with increasing O_2_ diffusion rate. According to the *K*–*L* equation [56], the calculated electron transfer number (*n*) is 3.8, which indicates that a four-electron process dominates the oxygen reduction for (Ni,Co)S_2_. Table S3 lists the ORR parameters of the three catalysts. The ORR path was further verified with a rotating ring-disk electrode (RRDE) at 1.3 V at a rate of 2 mV s^−1^. As shown in Fig. 4d, the *n* value (3.9) thus estimated is consistent with the result obtained from the *K*–*L* equation. It clearly indicates that the oxygen reduction proceeds via an efficient four-electron pathway. A comparison of the ORR performance of (Ni,Co)S_2_ with the performances of some of the reported catalysts is shown in Table S4. As a bifunctional electrocatalyst, the overall oxygen activity of (Ni,Co)S_2_ is evaluated by the potential difference (Δ*E* = *E*_*j*=10_–*E*_1/2_) between *E*_1/2_ for ORR and *E*_*j*=10_ for OER. In general, an efficient reversible oxidation reaction requires a small Δ*E*, with the Δ*E* of commercial state-of-the-art electrocatalysts reported as 0.94 V for Pt/C and 0.92 V for Ir/C and Ru/C [57]. Figure 4e shows that the Δ*E* of (Ni,Co)S_2_ is 0.79 V, which is much lower than those of the reported precious electrocatalysts (Table S5) as well as the Δ*E* of NiS_2_ (0.95 V) and CoS_2_ (0.94 V). This further indicates the excellent electrocatalytic characteristics of (Ni,Co)S_2_ as a multifunctional electrocatalyst.

**Fig. 5:**
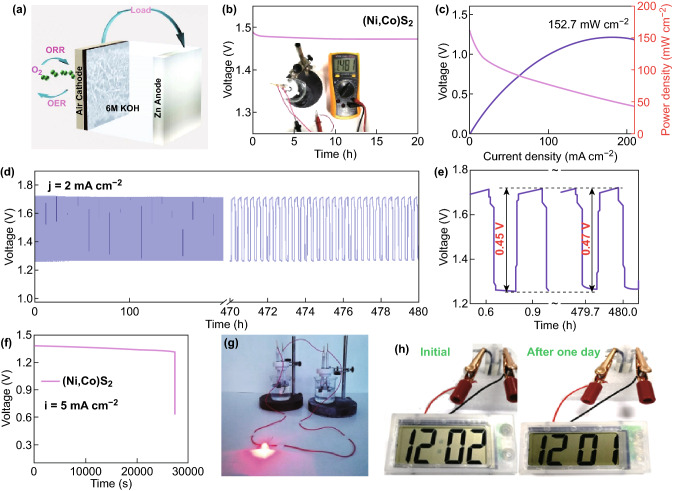
**a** Schematic illustration of rechargeable Zn–air battery. **b** Open cell voltage curve and **c** polarization and power density curves of (Ni,Co)S_2_-based primary Zn–air battery. **d** Galvanostatic discharge–charge cycling curves at 2 mA cm^−2^ of the rechargeable Zn–air battery. **e** Charge–discharge efficiency of the Zn–air battery at the beginning and end. **f** Long-time discharge curves of (Ni,Co)S_2_-based Zn–air battery at 5 mA cm^−2^. **g** Photograph of red LED powered by two (Ni,Co)S_2_-based Zn–air batteries. **h** Photographs of an electronic watch powered by the (Ni,Co)S_2_-based Zn–air battery

To demonstrate the application potential of (Ni,Co)S_2_ nanosheets as a bifunctional electrocatalyst for a Zn–air battery, we first constructed primary Zn–air batteries by using (Ni,Co)S_2_ as the electrocatalyst. The schematic diagram of a two-electrode liquid rechargeable battery is shown in Fig. 5a. The (Ni,Co)S_2_-based Zn–air battery shows an open cell voltage of 1.48 V at the beginning (Fig. 5b), which is similar to the result obtained by the multimeter test. After a continuous discharge of 20 h, the discharge voltage of (Ni,Co)S_2_ remains at 1.47 V, which is an ideal and stable high discharge voltage, whereas the open cell voltages of NiS_2_- and CoS_2_-based Zn–air batteries are 1.42 and 1.38 V, respectively (Fig. S18). As shown in Fig. 5c, the battery energy density of (Ni,Co)S_2_-based Zn–air battery is 152.7 W cm^−2^. A current density of 170 mA cm^−2^ was measured at an overpotential of 0.40 V. The charge and discharge cycle tests of (Ni,Co)S_2_-based Zn–air batteries were performed with 20 min cycles (charging for 10 min and discharging for 10 min; Fig. 5d). At a current density of 2 mA cm^−2^, it shows a stable charging voltage of 1.71 V and a discharge voltage 1.26 V with a very small charge–discharge gap of 0.45 V, which increases to 0.46 V after 480 h (Fig. 5e), revealing its superb stability. This value is much higher than that of many of the reported catalysts (Table S6). Similarly, the charge and discharge cycle curves were tested at a current density of 6 mA cm^−2^ for 100 h (Fig. S19). The results indicate that the (Ni,Co)S_2_-based Zn–air battery has a large specific capacity of 842 mAh g_Zn_^−1^ at a current density of 5 mA cm^−2^ (Fig. 5f), which is larger than that of both NiS_2_ (732 mAh g_Zn_^−1^) and CoS_2_ (681 mAh g_Zn_^−1^) (Fig. S20). Besides, two liquid zinc–air batteries in series could power a red LED (Fig. 5g), while one liquid zinc–air battery could power an electronic watch successfully (Fig. 5h).

**Fig. 6:**
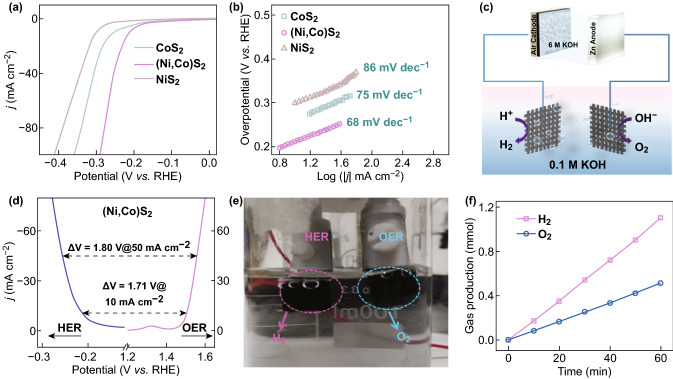
**a** HER polarization curves of (Ni,Co)S_2_, NiS_2_, and CoS_2_ at 5 mV s^−1^ in 0.1 M KOH. **b** Tafel slopes obtained from their polarization curves. **c** Schematic diagram of the self-assembled water-splitting system. **d** LSV curves of overall water splitting by (Ni,Co)S_2_ electrocatalyst in a two-electrode configuration at a scan rate of 5 mV s^−1^. **e** Two electrodes after water splitting powered by two in-series (Ni,Co)S_2_-based Zn–air batteries. **f** Time dependence of the mole quantities of H_2_ and O_2_ produced in the self-driven overall water-splitting unit

We also characterized the electrocatalytic HER properties of the three samples. As shown in Fig. 6a, the onset overpotential of (Ni,Co)S_2_ is 180 mV, which is lower than that of NiS_2_ (~ 264 mV) and CoS_2_ (~ 237 mV). When the current density reaches 10 mA cm^−2^, NiS_2_ and CoS_2_ require potentials of 298 and 254 mV, respectively, while (Ni,Co)S_2_ only requires 210 mV. In addition, the Tafel slope is 68 mV dec^−1^ for (Ni,Co)S_2_, which is smaller than that of NiS_2_ and CoS_2_ (Fig. 6b). Furthermore, (Ni,Co)S_2_ shows the largest *C*_dl_ of 40 mF cm^−2^ among the three samples and an excellent stability of more than 80,000 s for HER (Fig. S21, Table S7). Similarly, the HER polarization curve of CoS_2_ + NiS_2_ is given in Fig. S22. Unsurprisingly, the physically mixed sample CoS_2_ + NiS_2_ demonstrates an intermediate electrocatalytic hydrogen evolution efficiency. We also observed that (Ni,Co)S_2_ exhibits the best electrocatalytic properties among the three samples. The excellent reversibility makes it a very promising multifunctional catalyst. Furthermore, we used it as both cathode and anode to fabricate a water-splitting device. Figure 6c shows the schematic diagram of the self-assembled water-splitting unit powered by the (Ni,Co)S_2_-based Zn–air battery. In this self-assembled device, the anode undergoes oxidation reaction to generate oxygen, and the cathode undergoes reduction reaction to produce hydrogen. As shown in Fig. 6d, the LSV curves of the overall water-splitting reactions of (Ni,Co)S_2_ show a Δ*V* (*E*_OER_–*E*_HER_) of 1.71 and 1.80 V at 10 and 50 mA cm^−2^, respectively, revealing a noticeable electrocatalytic performance of (Ni,Co)S_2_ in a water-splitting energy installation compared to many reported catalysts (Table S8). Figure 6e shows that the cathode reacts to produce hydrogen and the anode oxidizes to produce oxygen, and the H_2_ and O_2_ were collected by the drainage method. After 60 min of continuous reaction, the amounts of H_2_ and O_2_ collected were 1.15 mmol and 0.57 mmol (the ratio is 2:1), respectively. The output voltage stability of the self-assembled device was tested using a multimeter (Fig. S23). It can be seen that the output voltage stabilized after 1 h and did not reduce in the next hour, which reveals its good stability.

## Conclusions

In summary, single-phase bimetallic (Ni,Co)S_2_ nanosheets were successfully synthesized by a hydrothermal route followed by thermal conversion to sulfide. With the purposely tuned nanosheet morphology, electronic structure, enhanced electrical conductivity, and active sites in the bimetallic sulfides, the (Ni,Co)S_2_ nanosheets demonstrated a superior electrocatalytic performance for oxygen evolution, oxygen reduction, and hydrogen evolution in an alkaline electrolyte. First principle calculation results indicate that the adsorption of HO^−^ to form *OOH on the (Ni,Co)S_2_ surface is the potential limiting step in the OER. When used as an electrode in a Zn–air battery, it demonstrated a small charge/discharge voltage gap of 0.45 V at 2 mA cm^−2^, a high peak power density of 153.5 mW cm^−2^, a specific capacity of 842 mAh g_Zn_^−1^ at 5 mA cm^−2^, and excellent cycling stability even after 480 h. The high efficiency demonstrates the application potential of the rechargeable Zn–air battery in powering an electrochemical water-splitting unit made of the same (Ni,Co)S_2_ nanosheets as both the electrodes, which exhibited a low cell voltage of 1.71 V at 10 mA cm^−2^. This work is helpful for improving the Zn–air battery performance and the utilization of new energy in the future.

## Electronic Supplementary Material

Below is the link to the electronic supplementary material.

Supplementary material 1 (PDF 1149 kb)
