# Supplementary material for: Bimetallic Nickel Cobalt Sulfide as Efficient Electrocatalyst for Zn–Air Battery and Water Splitting
Source: Nanomicro Lett. 2019 Jan 9;11:2. doi: 10.1007/s40820-018-0232-2 (PMC6325096; doi:10.1007/s40820-018-0232-2)
Supplement: Supplementary file 1 — Supplementary material 1 (PDF 1149 kb) [file 40820_2018_232_MOESM1_ESM.pdf]

Supporting Information for

# Bimetallic Nickel Cobalt Sulfide as Efficient Electrocatalyst for Zn-Air Battery and Water Splitting

Jingyan Zhang[1, ‡], Xiaowan Bai[2, ‡], Tongtong Wang[1], Wen Xiao[3], Pinxian Xi[4], Jinlan Wang[2], Daqiang Gao[1, *], John Wang[3, *]

[1]Key Laboratory for Magnetism and Magnetic Materials of MOE, Key Laboratory of Special Function Materials and Structure Design of MOE, Lanzhou University, Lanzhou 730000, People's Republic of China

[2]School of Physics, Southeast University, Nanjing 211189, People's Republic of China

[3]Department of Material Science and Engineering, National University of Singapore, Engineering Drive 3, 117575, Singapore

[4]Key Laboratory of Nonferrous Metal Chemistry and Resources Utilization of Gansu Province and The Research Center of Biomedical Nanotechnology, Lanzhou University, Lanzhou 730000, People's Republic of China

*Corresponding authors.

Email: gaodq@lzu.edu.cn (Daqiang Gao); msewangj@nus.edu.sg (John Wang)

‡Both authors contribute equally to this work

## S1 Materials Characterizations

The crystal structure of each sample was studied by using X-ray diffraction for phase analysis (XRD, X' Pert PRO PHILIPS with Cu Kα radiation). X-ray photoelectron spectroscopy (XPS, Kratos Axis Ultra) was conducted to study the elementary composition and the bonding characteristics in each sample. The morphology and high-resolution images were characterized using scanning electron microscopy (SEM, Hitachi S-4800) and transmission electron microscopy (TEM, TecnaiTM G2 F30, FEI, USA). Raman spectra were acquired using a Jobin-Yvon LabRam HR80 spectrometer (Horiba Jobin Yvon, Inc.) with 532 nm line of Torus 50 mW diode-pumped solid-state laser under backscattering geometry. Electrochemical measurements were performed in a standard three-electrode electrochemical cell using an electrochemical workstation (CHI660e).

## S2 Preparing of Glassy Carbon Electrode and Rotating Ring-Disk Electrode

The electrode liquid is coated to glassy carbon electrode or rotating ring-disk electrode with a pipette with a capacity of 0.2 mg $cm^{-2}$. The coated electrode were let dried in air before test.

## S3 Calculation Details

The DFT calculations were performed by Vienna ab initio simulation package (VASP). The standard generalized-gradient approximation (GGA) in the form of the Perdew-Burke-Ernzerhof (PBE) exchange model was used. The energy cutoff for the plane-wave basis set and the convergence threshold to obtain the wave functions were 400 eV and $10^{-5}$ eV, respectively. 3d electrons of Ni was treated using the GGA+U method with the $U_{eff}$ (U-J) of 5.76 eV. Ionic relaxations were conducted until all force components became <0.02 eV $Å^{-1}$. For the density of states (DOS), the Brillouin zone is represented by the set of 5×5×5 k points for the geometry optimizations. A rectangular supercell with 11.00 Å×11.00 Å was used to calculate the OER activity with the active site on (100) surface. In alkaline environment, the standard oxygen reduction reaction is described as follow [S1]:

$$O_2\ (g) + H_2O(l) + 4e^- \rightarrow 4OH^-$$

$$E^0 = 0.402\ V$$

Four steps are involved in the OER performance, the corresponding Gibbs free energy (G) was valued via the following mechanism:

**Step 1**:

$$OH^- + * \rightarrow OH^* + e^-$$

$$\Delta G_1 = \left[\left(E_{DFT}^{OH^*} + ZPE - TS^0\right) - E_{DFT}^{*}\right] + 9.952\ Ev$$

**Step 2:**

$$OH^- + OH^* \rightarrow O^* + H_2O(l) + e^-$$

$$\Delta G_2 = \left[\left(E_{DFT}^{O^*} + E_{DFT}^{H_2O(g)}\right) - E_{DFT}^{OH^*} + \left(\Delta ZPE - TS^0\right)\right] + 9.952\ Ev$$

**Step 3:**

$$OH^- + O^* \rightarrow OOH^* + e^-$$

$$\Delta G_3 = \left[\left(E_{DFT}^{OOH^*} - E_{DFT}^{O^*}\right) + \left(\Delta ZPE - TS^0\right)\right] + 9.952\ Ev$$

**Step 4:**

$$OH^- + OOH^* \rightarrow * + O_2\,(g) + H_2O(l) + e^-$$

$$\Delta G_4 = (E^*_{DFT} + \left(4.92 + 2\left(E_{DFT}^{H_2O(g)} - E_{DFT}^{H_2(g)}\right) + E_{DFT}^{H_2O(g)}\right) - E_{DFT}^{OOH^*} + (\Delta ZPE - TS^0) + 9.952\ \text{eV}$$

The Gibbs free energy (G) for OER is

$$G^{OER} = \max[\Delta G_1^0, \Delta G_2^0, \Delta G_3^0, \Delta G_4^0]$$

The theoretical onset overpotential is

$$\eta = \max[\Delta G_1^0, \Delta G_2^0, \Delta G_3^0, \Delta G_4^0]/e - 0.402\ \text{V}$$

Where ZPE is zero point energy and TS is entropic contributions (TS) to the free energies.

## S4 HER Measurements

In the standard three-electrode electrochemical cell of electrochemical workstation (CHI660e), graphite electrode, Ag/AgCl, and glassy carbon electrode were used as the counter, reference, and working electrode. All the data were recorded at a sweep rate of 5 mV $s^{-1}$ after applying a number of cyclic voltammetric scannings until they were stable. Current density was normalized to the geometrical area of the working electrode. The electrochemical measurements were *iR*-corrected until otherwise specified. The potential of Ag/AgCl is related to RHE by the equation of *E*(RHE(V))= E(Ag/AgCl) + 0.197 V+ 0.059*pH.

## S5 OER Measurements

For OER characterization, the typical process is similar to that of HER, except that the test was conducted in an alkaline electrolyte 0.1 M KOH.

## S6 ORR Measurements

The catalyst ink was pipetted onto the disk electrode or ring disk electrode to obtain a catalyst loading of 0.2 mg $cm^{-2}$, which was used to test for ORR. Electrochemical experiments were carried out in $O_2$-saturated 0.1 M KOH electrolyte for ORR. The potential range is cyclically scanned between 0.2 and 1.0 V *vs.* RHE with a scan rate of 2 mV $s^{-1}$. The CV and LSV were obtained at the ambient temperature after purging with $O_2$ or $N_2$ gas for 30 min. The potential cycling was repeated until stable voltammogram curves were obtained. RDE measurements were made at rotating rates varying from 400 to 2400 rpm, at a scan rate of 2 mV $s^{-1}$.

Kinetic parameters were obtained on the basis of the following Koutecky–Levich (K-

L) equation:

$$1/j=1/j_k+1/(B\omega^{1/2})$$

*B*, the slope of *K-L* plot, can be obtained from the following:

$$B=0.2\ nF(D_{O_2})^{2/3}v^{-1/6}C_{O_2}$$

where $j$ is the measured current density, $j_k$ is the kinetic current density, $\omega$ is the rotation speed (the constant of 0.2 is used when the rotation speed is expressed in rpm), $n$ is the electron transfer number, $F$ is the Faraday constant (96485 C mol$^{-1}$), $C_{O_2}$ is the saturated concentration of $O_2$ in the electrolyte ($1.21\times10^{-6}$ mol cm$^{-3}$), $D_{O_2}$ is the diffusion coefficient of $O_2$ in 0.1 M KOH solution ($D_{O_2}$=$1.9\times10^{-5}$ cm$^2$ s$^{-1}$), $v$ is the kinetic viscosity of the electrolyte (0.01 cm$^2$ s$^{-1}$).

**S7 Zn–air Batteries**

Home-made electrochemical cells of rechargeable Zn–air battery are constructed in the present work. The active material, made as detailed in the experimental procedure, was coated on carbon paper substrate as the air cathode. A polished Zn plate was employed as the anode, and a 6 M KOH + 0.2 M $Zn(Ac)_2$ aqueous solution was utilized as the electrolyte. Battery tests were performed at room temperature using a LAND CT2001A instrument. In the cycling test, one cycle typically consists of one discharging step (2 mA cm$^{-2}$ for 5 min) followed by one charging step of the same current density and duration time.

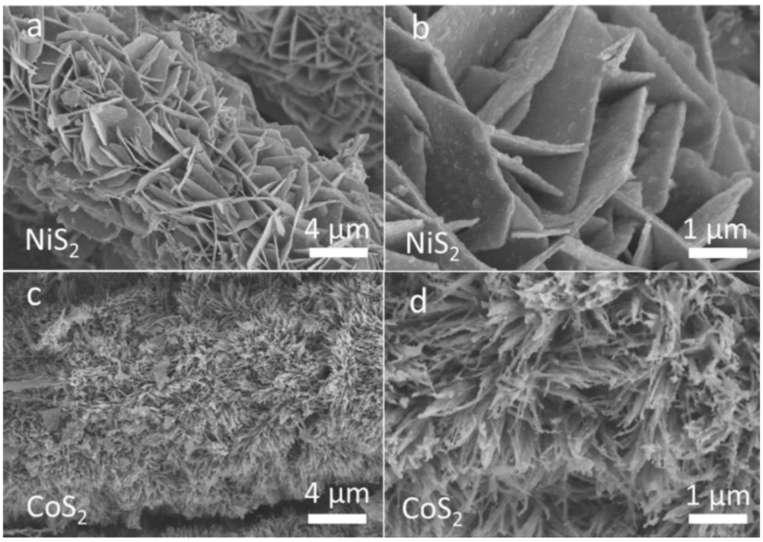


**Fig. S1** **a** Low-magnification, and **b** high-magnification SEM image of $NiS_2$. **c** Low-magnification and **d** high-magnification SEM image of $CoS_2$

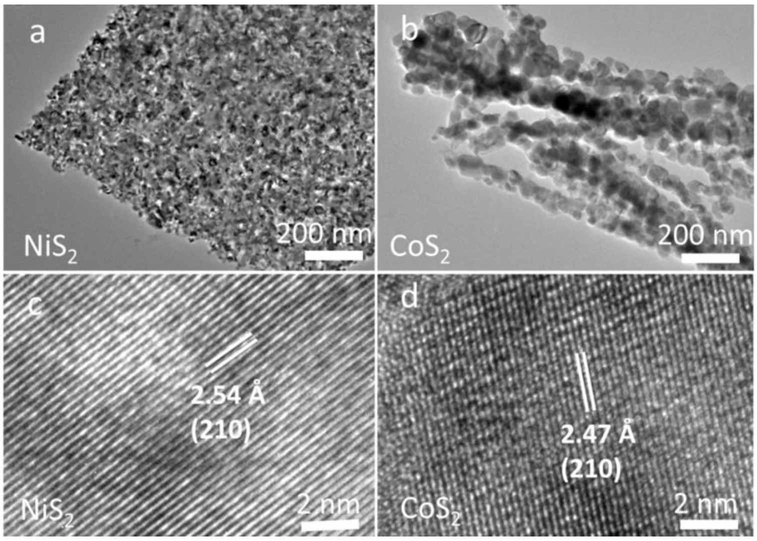


**Fig. S2** TEM images of **a** $NiS_2$, and **b** $CoS_2$. High resolution TEM images of **c** $NiS_2$ and **d** $CoS_2$

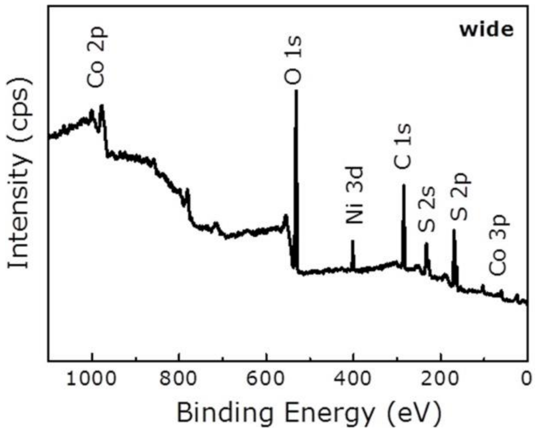


**Fig. S3** The wide spectrum of XPS of $(Ni,Co)S_2$

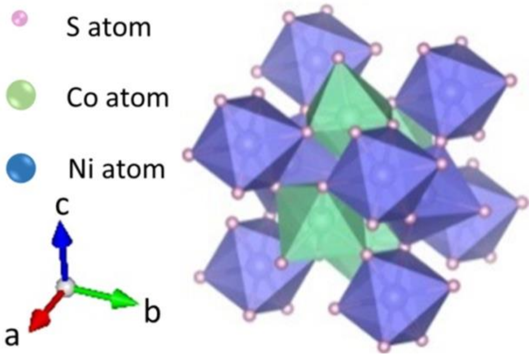


**Fig. S4** Schematic diagram of atomic structure of $(Ni,Co)S_2$

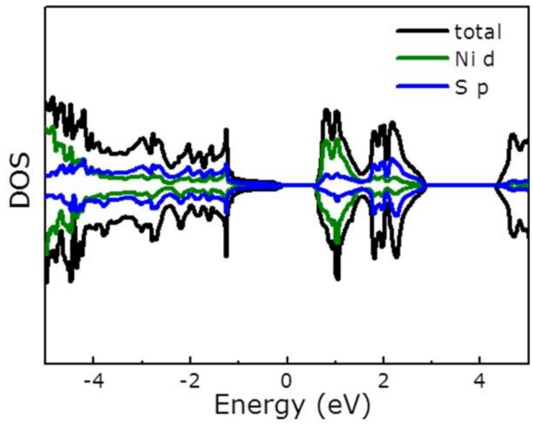


**Fig. S5** Calculated partial density of states (PDOS) result for $NiS_2$

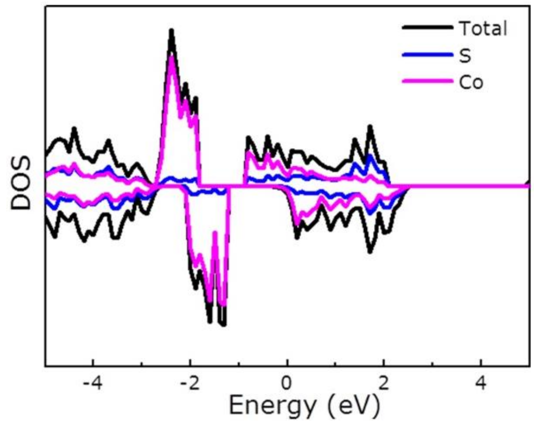


**Fig. S6** Calculated partial density of states (PDOS) result for $CoS_2$

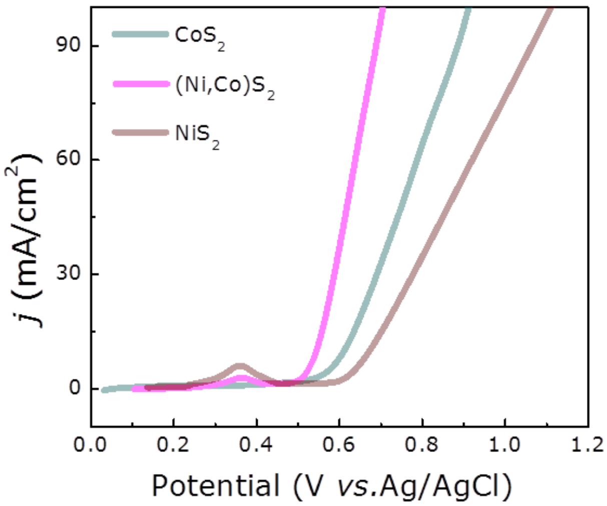


**Fig. S7** Linear voltammetry scanning (LSV) curves (*vs.*Ag/AgCl) of $(Ni,Co)S_2$, $NiS_2$, $CoS_2$ and Ir/C (20% Ir) at 5 mV/s in 0.1 M KOH

## S8 Calculation of Effective Active Surface Area (ECSA)

The double layer capacitance ($C_{dl}$) is obtained by cyclic voltammetry at different scan rates (in the range of 20~180 mV $s^{-1}$) to be linearly proportional to effective active surface area (ECSA). The potential is in the range from 0.2 to 0 V *vs.* Ag/AgCl, the $C_{dl}$ is estimated by plotting the difference between anodic and cathodic currents ($j_a$-$j_c$) at 0.1 V *vs.* Ag/AgCl against various scan rates, where the slope is double $C_{dl}$. The specific capacitance $C_{dl}$ can be converted into an electrochemical active surface area (ECSA) using the specific capacitance value for a flat standard with 1 $cm^2$ of real surface area. The specific capacitance for a flat surface is generally found to be in the range of 20-60 μF $cm^{-2}$ [S2]. In the following calculations of ECSA we assume 40 μF $cm^{-2}$,

$$\mathrm{ECSA} = \frac{C_{dl}}{40\ \mu F\ cm^{-2}\ per\ cm^2_{ECSA}}$$

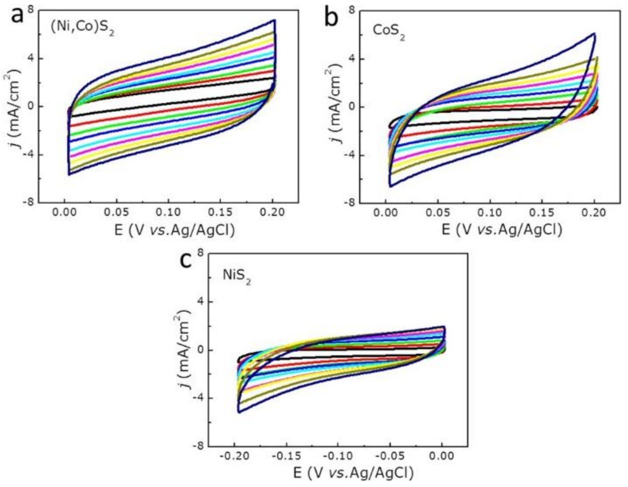


**Fig. S8** CV curves of **a** $(Ni,Co)S_2$, **b** $CoS_2$ and **c** $NiS_2$ in 0.1 M KOH solution

## S9 Turnover Frequency (TOF) in OER Calculation [S3]

The TOF values were calculated by assuming that every metal atom is involved in the catalysis:

$$\mathrm{TOF} = jS/(4Fn)$$

Here, $j$ (mA $cm^{-2}$ ) is the measured current density, S is the surface area of the electrode, the number 4 means 4 electrons per mol of $O_2$, F is the Faraday's constant (96,485 C $mol^{-1}$ ) and n is the moles of coated metal atom on the electrode.

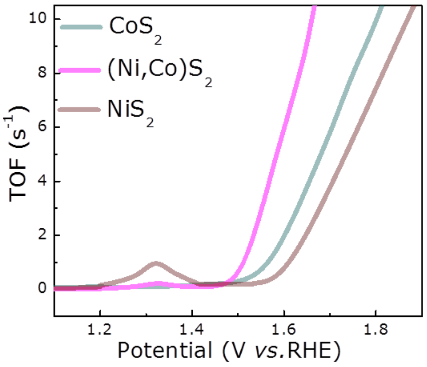


**Fig. S9** The OER TOF results of $(Ni,Co)S_2$, $NiS_2$ and $CoS_2$

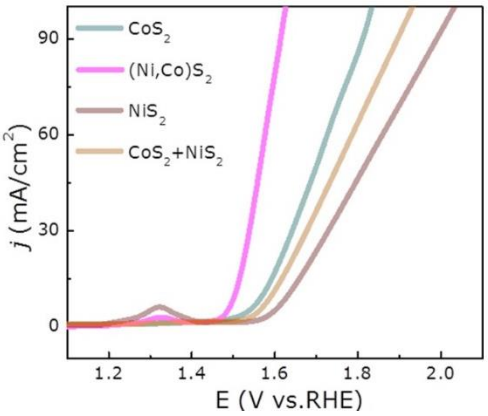


**Fig. S10** Polarization curves of $(Ni,Co)S_2$, $NiS_2$, $CoS_2$ and $(CoS_2+NiS_2)$ at 5 mV $s^{-1}$ in 0.1 M KOH

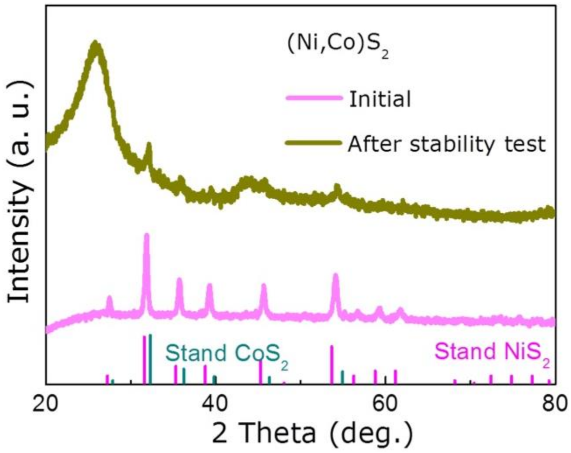


**Fig. S11** XRD patterns of $(Ni,Co)S_2$ at the initial stage and after the stability test

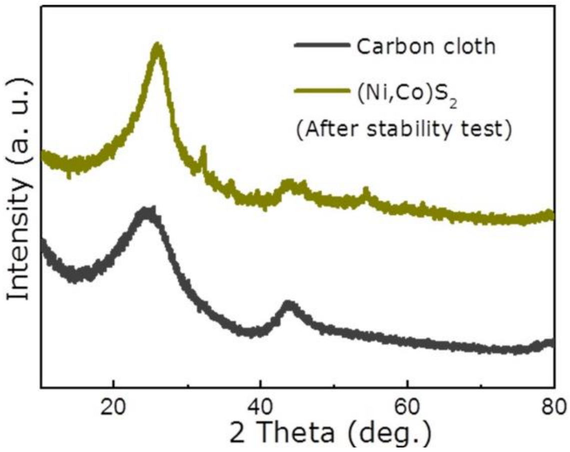


**Fig. S12** XRD patterns of carbon cloth, and (Ni,Co)$S_2$ after the stability test

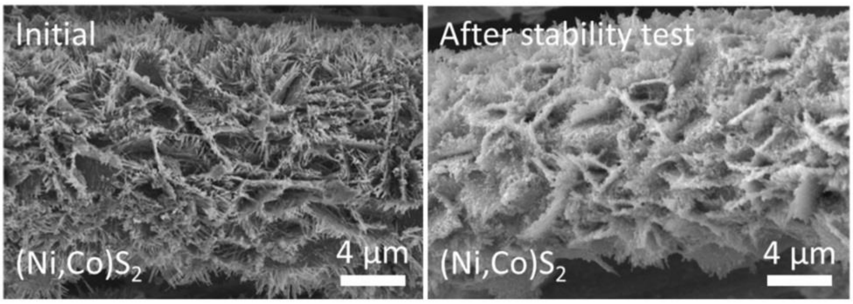


**Fig. S13** SEM image of **a** initial, and **b** after the stability test of (Ni,Co)$S_2$

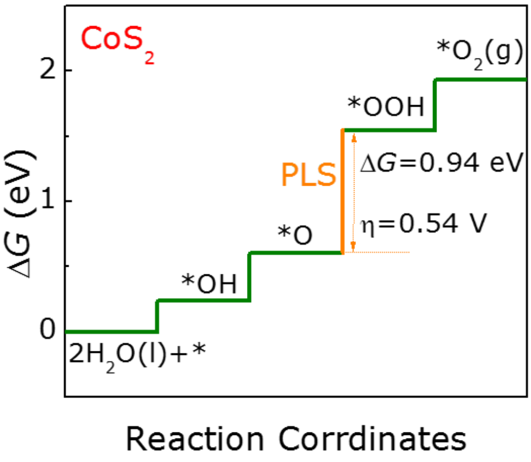


**Fig. S14** Schematic of the Gibbs free energy changes for the four elementary steps during the OER on $CoS_2$ (100) surface

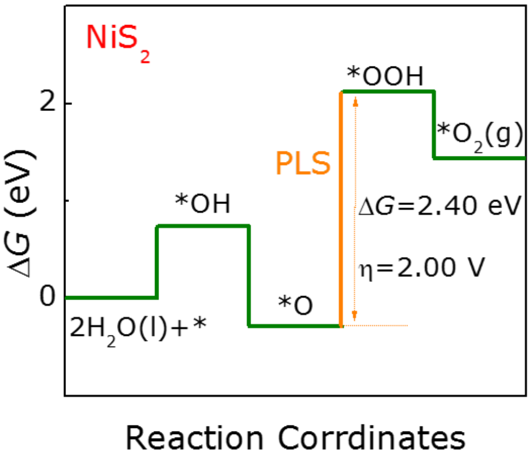


**Fig. S15** Schematic of the Gibbs free energy changes for the four elementary steps during the OER on $NiS_2$ (100) surface

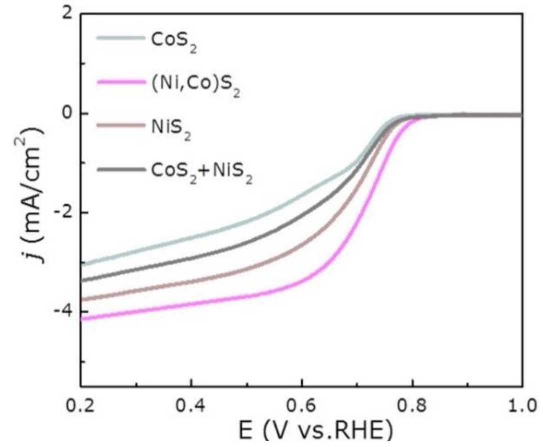


**Fig. S16** ORR polarization curves of $(Ni,Co)S_2$, $NiS_2$, $CoS_2$ and $(CoS_2+NiS_2)$ at 2 mV $s^{-1}$ in 0.1 M KOH at 1600 rpm

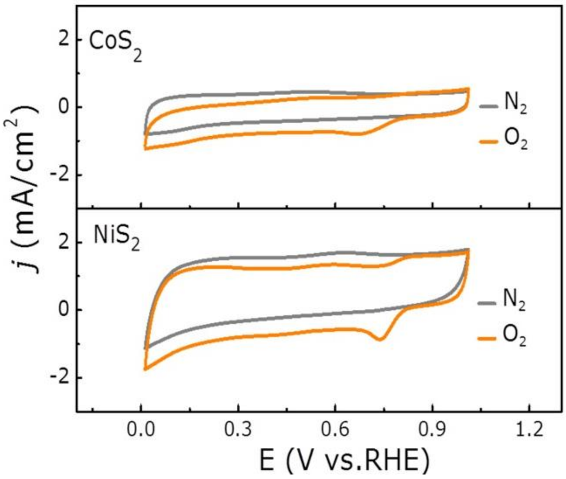


**Fig. S17** CVs of $NiS_2$ and $CoS_2$ in $O_2$ and $N_2$-saturated 0.1 M KOH solution

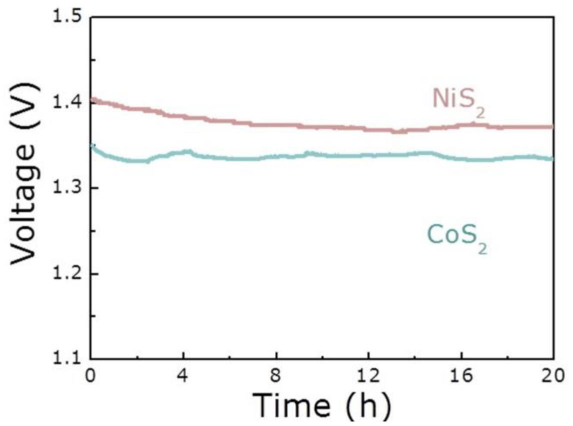


**Fig. S18** Open cell voltage curves of of $NiS_2$ and $CoS_2$

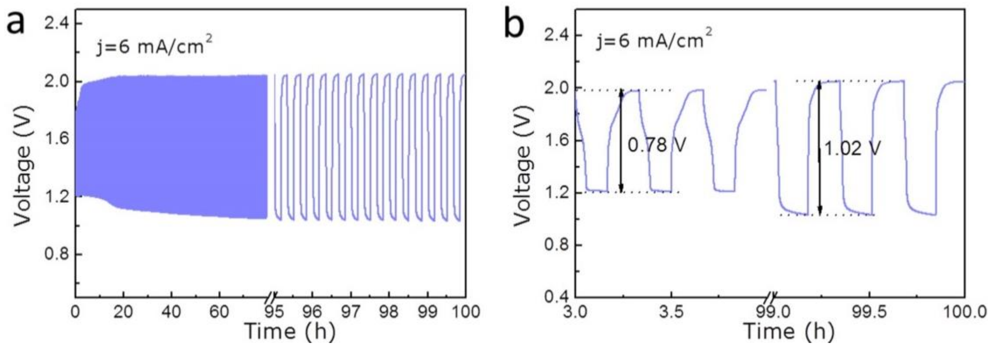


**Fig. S19 a** Galvanostatic discharge-charge cycling curves at 6 mA $cm^{-2}$ of the rechargeable Zn-air battery. **b** Charge discharge efficiency at the beginning and final of Zn-air battery at 6 mA $cm^{-2}$

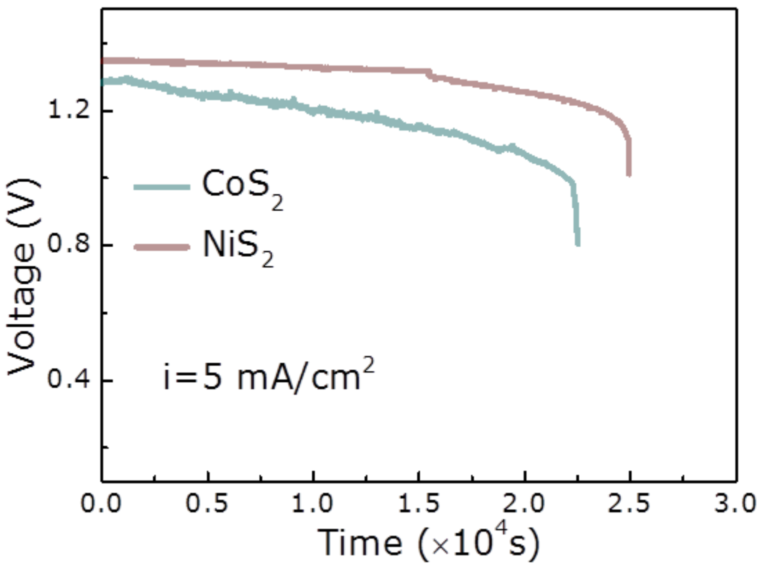


**Fig. S20** Long-time discharge curves of $NiS_2$ and $CoS_2$ Zn-air battery at 5 mA $cm^{-2}$

## S10 Turnover Frequency (TOF) in HER Calculation

Voltammetric charges (Q) is calculated by the following equation [S4]:

$$Q = 2Fn$$

Where F is Faraday constant (96,480 C $mol^{-1}$), n is the number of active sites. The factor 2 suggests that the formation of one hydrogen molecule needs two electrons in HER. In the experiment, the voltammetry curve is obtained by CV measurements with phosphate buffer (pH = 7) at a scan rate of 50 mV $s^{-1}$. When the number of voltammetric (Q) is obtained after deduction of the blank value.

The turnover frequency (TOF) can be calculated with the following equation:

$$TOF = I/Q$$

Where $I$(A) is the current of the polarization curve, we obtained it from the LSV measurements.

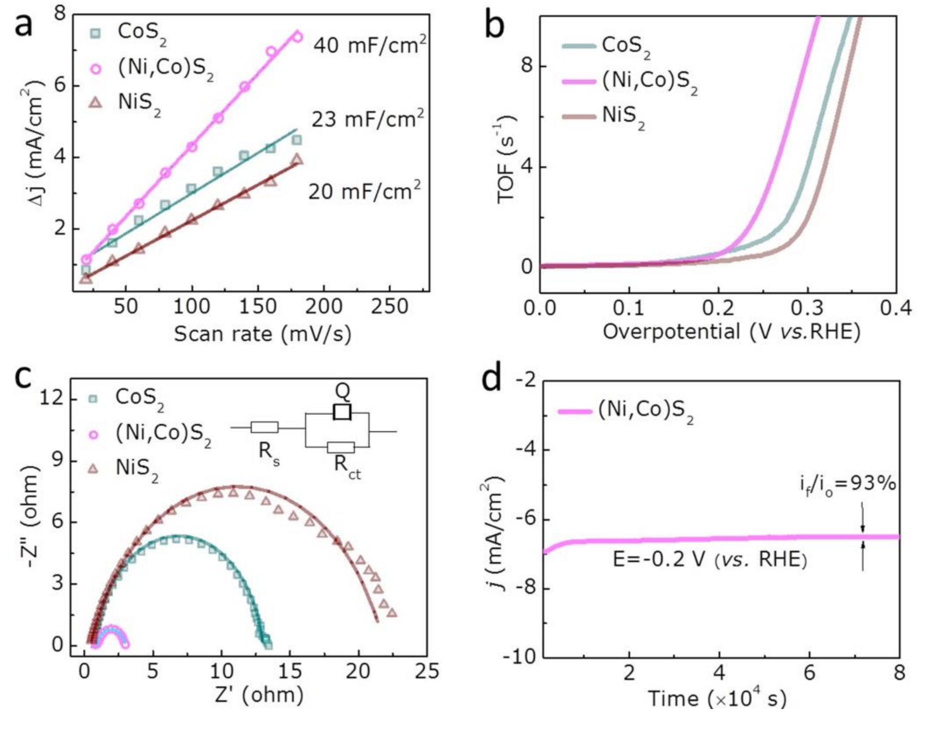


**Fig. S21 a** The $C_{dl}$ obtained via cyclic voltammetry at different scan rates. **b** The TOF of $(Ni,Co)S_2$, $NiS_2$ and $CoS_2$. **c** EIS of $(Ni,Co)S_2$, $NiS_2$ and $CoS_2$, the insert is analogue circuit diagram. **d** The *i-t* curve of $(Ni,Co)S_2$ at -0.2V *vs*.RHE for $8\times10^4$ s

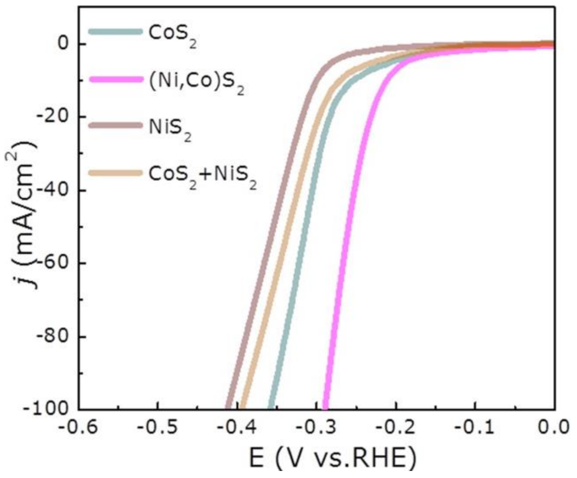


**Fig. S22** HER polarization curves of (Ni,Co)$S_2$, $NiS_2$, $CoS_2$ and ($CoS_2$+$NiS_2$) at 5 mV $s^{-1}$ in 0.1 M KOH

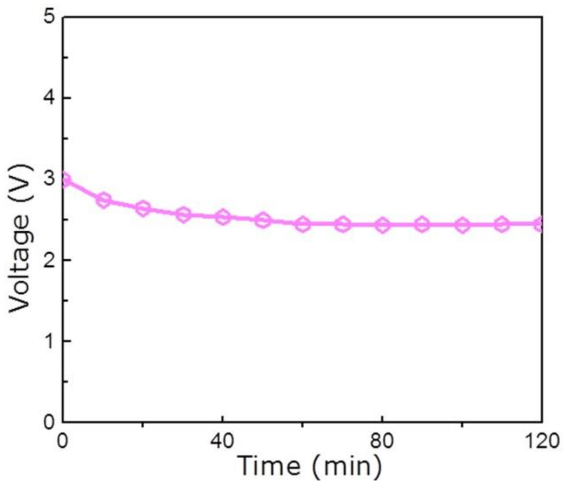


**Fig. S23** Time dependence of the Voltage measured by multimeter in the self-driven overall water-splitting unit

**Table S1** Comparison of OER performance for catalysts studied and Ir/C

| Catalysts | Onset potential [V *vs.* RHE] | $\eta$ at $J$ = 10 mA $cm^{-2}$ [mV] | Tafel slope [mV dec] | $C_{dl}$ [mF $cm^{-2}$] | TOF at η=1.55 V [$s^{-1}$] |
|---|---|---|---|---|---|
| (Ni,Co)$S_2$ | 240 | 270 | 58 | 41 | 3.02 |
| $NiS_2$ | 340 | 410 | 123 | 16 | 0.22 |
| $CoS_2$ | 270 | 350 | 107 | 30 | 0.65 |
| Ir/C | 250 | 310 | 77 | - | - |

**Table S2** Comparison of OER performance for previous catalysts

| Catalysts | Onset potential [V *vs.* RHE] | η at J = 10 mA $cm^{-2}$ [mV] | Tafel slope [mV/dec] | Stability [h] | References |
|---|---|---|---|---|---|
| **(Ni,Co)$S_2$** | **240** | **270** | **58** | **19.4** | **This work** |
| CoNi LDH/CoO-1 | 250 | 300 | 123 | 36 | [S5] |
| NG-NiCo | 350 | - | 614 | 12 | [S6] |
| $Ni_xCo_{3-x}O_4$ NWs | 500 | - | 59 | - | [S7] |
| CoNi$(OH)_x$ | 250 | 280 | 77 | 24 | [S8] |
| NiCoFe-LDH | - | 265 | 98 | 30 | [S9] |
| $NiCoMnO_4$/N-rGO | 270 | 520 | 128 | 3 | [S10] |
| $NiCo_2O_4$/NF | - | - | 135 | 10 | [S11] |
| (NiCo)S/OH | - | 227 | 77.5 | - | [S12] |
| Fe-$NiCo_2O_4$ | 245 | 302 | 42 | 10 | [S13] |
| $Ni_{0.5}Co_{0.5}S_2$ | - | - | 61 | 4 | [S14] |
| $Ni_{0.13}Co_{0.87}S_{1.097}$ | 262 | 316 | 54.7 | 2 | [S15] |

**Table S3** Comparison of ORR performance for catalysts studied

| Catalysts | Onset potential [V *vs.* RHE] | Half-wave potential $E_{1/2}$ [V *vs.* RHE] | Cathodic peak at $O_2$-saturated [V *vs.* RHE] | Limiting current density [mA $cm^{-2}$] | Electron transfer number (*n*) |
|---|---|---|---|---|---|
| (Ni,Co)$S_2$ | 0.82 | 0.71 | 0.75 | 4.2 | 3.98 |
| $NiS_2$ | 0.76 | 0.68 | 0.74 | 3.7 | 2.21 |
| $CoS_2$ | 0.79 | 0.63 | 0.67 | 3.2 | 2.14 |
